# A Double-Blind, Randomized, Placebo-Controlled Trial of Heat-Killed *Pediococcus acidilactici* K15 for Prevention of Respiratory Tract Infections among Preschool Children

**DOI:** 10.3390/nu12071989

**Published:** 2020-07-03

**Authors:** Haruka Hishiki, Tadaomi Kawashima, Noriko M. Tsuji, Naho Ikari, Ryo Takemura, Hiroshi Kido, Naoki Shimojo

**Affiliations:** 1 Department of Pediatrics, Chiba University Hospital, Chiba 260-8670, Japan; hishikih@faculty.chiba-u.jp; 2 Research and Development Division, Kikkoman Corporation, Chiba 278-0037, Japan; takawashima@mail.kikkoman.co.jp (T.K.); nikari@mail.kikkoman.co.jp (N.I.); 3 Biomedical Research Institute, National Institute of Advanced Industrial Science and Technology, Ibaraki 305-0046, Japan; nm-tsuji@aist.go.jp; 4 Clinical Research Center, Chiba University Hospital, Chiba 260-8677, Japan; rtakemura@keio.jp; 5 Clinical and Translational Research Center, Keio University Hospital, Tokyo 160-8582, Japan; 6 Institute for Enzyme Research, Tokushima University, Tokushima 770-8503, Japan; kido@tokushima-u.ac.jp; 7 Center for Preventive Medical Sciences, Chiba University, Chiba 263-8522, Japan

**Keywords:** *Pediococcus acidilactici* K15, double-blind study, preschool children, respiratory tract infections, secretory IgA, febrile days, safety

## Abstract

Although some probiotic bacteria have been reported to prevent infections in children, there are few well-designed double-blind studies. Here we evaluated the effects of a probiotic strain of lactic acid bacteria (LAB), *Pediococcus acidilactici* K15, on viral respiratory tract infections in preschool children. A four-month, randomized, double-blind, placebo-controlled study was performed in 172 healthy children aged 3 to 6 years. Subjects were administered dextrin alone or dextrin including heat-killed K15 (5 × 10^10^ bacteria). The number of febrile days was the primary outcome. The number of absent days from preschools and the influenza incidence were secondary outcomes. Secretory IgA (sIgA) concentrations in saliva were measured as an exploratory outcome. The primary and secondary outcomes were not significantly different between both groups. Analyses in children with little intake of fermented foods including LAB showed that the duration of a fever significantly decreased by K15 intake. The salivary sIgA level in the K15 group was maintained significantly higher than it was in the placebo group. The effects of K15 on preventing viral respiratory tract infections were not observed without the restriction of fermented foods intake. However, K15 supported anti-infectious immune systems in children who took less fermented foods and the maintenance of salivary sIgA levels in all subjects.

## 1. Introduction

In modern societies, many children usually spend much time in preschools, nursery schools or kindergartens. This means that they have many opportunities to be exposed to infectious pathogens, especially viruses. Some vaccines, such as influenza and pneumococcal vaccines, are effective in preventing childhood infections, however their effectiveness is limited, particularly for the common cold [1,2,3]. Therefore, it is essential for these children to properly gain the immunological capacity to recognize and respond against pathogens in order to prevent infectious diseases.

Lactic acid bacteria (LAB) are utilized for fermented food to both prolong the preservation period by lowering pH and producing bacteriocins [4,5] and to produce a variety of flavors [6]. They are also known to have a variety of beneficial effects on human health [7]. Recently, it was revealed that some probiotic strains of LAB activate the innate immune system and then activate the acquired immune system, resulting in protection from immune diseases and infectious diseases [8,9,10,11,12,13]. Some of these were reported to induce the production of type I interferons (IFNs), IFN-α and IFN-β from dendritic cells (DCs), which play an important role in anti-viral effects [8,10]. IFN-β is secreted by myeloid DCs (mDCs), whereas IFN-α is secreted by plasmacytoid DCs (pDCs) [14,15]. Probiotic strains of LAB work on both DCs to secrete different kinds of type I IFNs and protect against viral infections [8,10].

Another major mechanism of LAB that enhances the host defense at mucosal sites, such as the gut, is the activation of the production of pathogen-specific or non-specific antibodies (Ab) for repelling pathogens [8,11,16,17]. Secretory IgA (sIgA) at mucosal sites such as the gut, oral cavity or respiratory tract protects from pathogen invasion by inhibiting the adherence of diverse and variable mucosal microorganisms [18].

It has been known that LAB have various kinds of probiotic effects on human health, such as the improvement of the gastrointestinal tract and the activation of immune functions [7,12,19]. Since cell wall components, such as peptidoglycan and lipoprotein, and nucleic acids in LAB contribute to the effects on immune functions [8,20,21,22], both live and heat-killed LAB are expected to activate immune responses and protect from immune diseases. In fact, some strains of heat-killed LAB were reported to have anti-allergic, anti-infectious and anti-inflammatory effects in humans and mice [8,9,10,20,23]. It should be noted that the oral administration of live LAB has some risks, such as diarrhea, bacterial translocation and the acquisition of antibiotic resistance genes, especially in children [24]. Moreover, raw dairy products must be stored at low temperatures and are perishable. On the other hand, heat-killed LAB can be used safely with little side effects [24] and are suitable to store for a long time at room temperature. Therefore, heat-killed LAB have advantages in their stability and safety benefits to manage the clinical trials.

In our previous study, a heat-killed probiotic strain, *Pediococcus acidilactici* strain K15, induced the production of IFN-β by human BDCA1^+^ DCs [25] and a large amount of IgA by human B cells stimulated with BDCA1^+^ DCs [19]. As K15 was expected to exert the inhibitory effects against viral respiratory tract infections from these results, we performed a randomized, placebo controlled, double-blind trial in preschool children who have high risks for infection.

## 2. Materials and Methods

### 2.1. Preparation of Clinical Test Foods

Heat-killed K15 powder was prepared by Kikkoman Corporation (Chiba, Japan). K15 was cultured using media including soy peptide, yeast peptone, glucose and sodium acetate for 24 h. After that, K15 was heat-killed at 90 °C, washed with a saline by using ultrafiltration columns and spray-dried with dextrin. The test foods for the K15 group were prepared by blending this K15 powder with an additional dextrin powder. For the placebo group, just a dextrin powder was packed. The test foods for the K15 group had 1 g of dextrin including 9.1 mg of heat-killed K15 (5 × 10^10^ bacteria), while that for the placebo group had 1 g of dextrin alone.

### 2.2. Clinical Study Design

We conducted a randomized, double-blind, placebo-controlled trial in 172 children who went to one of three preschools in Chiba (Japan), from November 2016 to February 2017. The subjects took test foods every day for sixteen weeks. The trial was conducted by Chiba University and was approved by the Ethics Committee of Chiba University (Chiba, Japan) (registration No. G28016) in compliance with the Declaration of Helsinki (2013). Written informed consent was obtained from the parents of the participants prior to the study. The trial was registered in the University Hospital Medical Information Network (UMIN) Clinical Trials Registry as UMIN000024432. All of the subjects’ parents reported in questionnaires about their family histories of allergic diseases and their school years. Further, every day they recorded the subject’s number of consumed items of food, including the other LAB, the consumption of test foods, as well as the number of vaccinations against the influenza virus and frequency of common cold symptoms in their family during the test period. These records were collected and checked once a week, followed by being transferred to the data center in Chiba University Hospital.

### 2.3. Outcomes

The number of febrile days (≥37.5 °C) during the test period was the primary outcome. Secondary outcomes were as follows: (a) the number of absent days from preschool due to common cold symptoms; (b) the incidence of influenza virus infections that were diagnosed by physician; (c) the duration of fevers during influenza virus infection; and (d) side effects associated with intake of K15. Salivary influenza virus-specific sIgA/IgG and total sIgA/IgG levels in samples collected before and after treatment were measured as exploratory outcomes.

### 2.4. Subjects for the Clinical Trial

The subjects were recruited from students in 3 preschools (Chiba, Japan) and 172 subjects participated in this clinical trial. All three preschools have three grades: the 1st year, the 2nd year and 3rd year with children aged 5–6, 4–5, and 3–4, respectively. Subjects were randomized and divided into two groups (85 in the placebo group and 87 in the K15 group) by DATATRAK ONE (DATATRAK International Inc., Mayfield Heights, OH, USA). The randomization scheme was generated by a statistician who did not have contact with study participants. The inclusion criteria were as follows: subjects who (a) were 3–6 years old and healthy, (b) went to preschool and (c) agreed to participate in this trial and provided informed consent from their parents. The exclusion criteria were as follows: subjects who (a) had an allergic reaction to products containing soy or LAB, (b) had congenital heart diseases or severe respiratory diseases, (c) were born preterm (<37 weeks’ gestation) or at low birth weight (<2500 g) and (d) were inappropriate cases for the trial as defined by physicians.

### 2.5. Determination of Salivary sIgA Secretion Rate and Concentration

Saliva samples were collected for the measurement of influenza virus-specific IgA/IgG and total IgA/IgG levels before and after treatment. Influenza virus-specific IgA/IgG concentrations were determined by ELISA, as described previously [26,27]. Total sIgA concentrations were measured using a human IgA quantitation kit (Bethy Laboratories, Montgomery, TX, USA) according to the instructions supplied by the manufacturer.

### 2.6. Statistical Analysis

The sample size was estimated to detect a one-third reduction in febrile days during the test period between the two groups. According to a preliminary survey, the average number of absent days due to fever during the same period was 1.2 days in these preschools. To achieve 90% power with a two-sided *p*-value < 0.05 as significant, it was estimated that at least 90 patients per group were required. The student’s t-test was used to compare continuous variables, whereas Fisher’s exact test was used to compare categorical data. The group comparison between the placebo group and the K15 group was evaluated by SAS 9.4. sIgA results were analyzed in a parametric method with the outliers excluded. The statistical significance between the two groups was determined with a two-tailed Student’s t-test for unpaired data with *p* values of < 0.05 considered significant.

## 3. Results

### 3.1. Participants’ Baseline Characteristics

**Figure 1:**
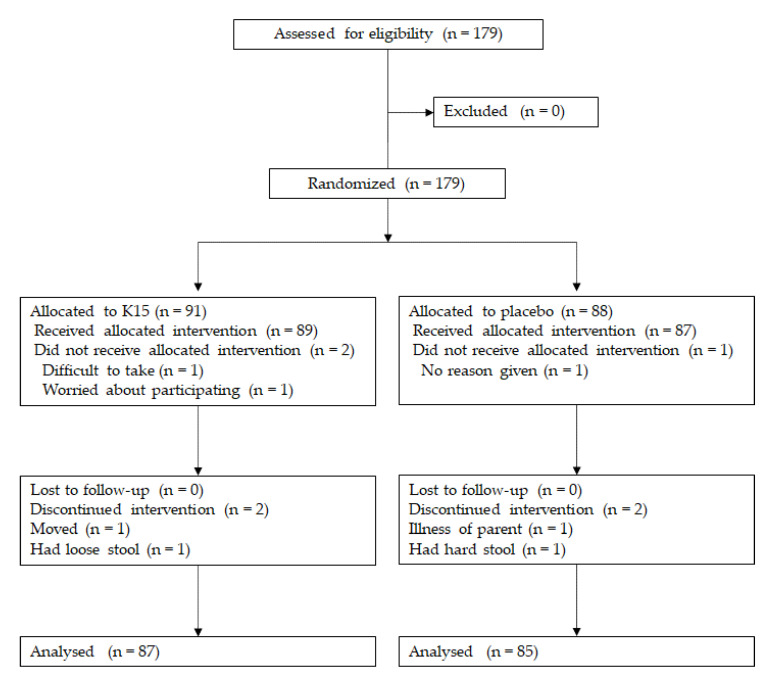
Flowchart of the study recruitment.

One hundred and seventy-nine participants were assessed for eligibility. As a result, no one was excluded due to not meeting eligibility. The 179 children who met the inclusion criteria were randomized. We further excluded four participants from the K15 group and three from the placebo group. Finally, 87 participants received K15 and 85 received a placebo (Figure 1). There was no bias in the participants’ background and the assignment was evenly implemented (Table 1).

### 3.2. Primary Outcome (Number of Febrile Days during the Exam Period)

The primary outcome is shown in Table 2. There was no significant difference between the placebo and K15 groups.

### 3.3. Secondary Outcomes

Some of the secondary outcomes were not significantly different between the two groups, such as the number of absent days from preschool, the incidence of influenza virus infections or the duration of fever during influenza virus infection (Table 3). As we obtained data for the amount of dietary intake, including other LAB, such as fermented foods or yogurt, the number of febrile days due to common cold symptoms were analyzed in children who took less fermented foods or yogurt (≤ 20 days during the test period). In this analysis, the number of febrile days in the K15 group was significantly less than that in the placebo group (*p* = 0.042) (Table 4). As for the safety of K15 intake, the number of adverse events was not different between the two groups (Table 3), indicating that heat-killed K15 was safe for children in this dosage.

### 3.4. Salivary sIgA

Regarding salivary antibodies, total sIgA in the K15 group was higher than that in the placebo group after the treatment, but we could not observe a significant difference between the two groups (*p* = 0.064) (Table 5). The level of total sIgA in the placebo group decreased during the test period, whereas K15 intake maintained a slight increase in the level. As a result, the change of the total sIgA during the test period was significantly higher in the K15 group (*p* = 0.044) (Table 5). Influenza virus-specific IgA or IgG was not different between the groups (data not shown), indicating that influenza virus morbidity might influence the levels of these specific antibodies.

## 4. Discussion

Many clinical studies have investigated the effects of probiotic LAB against respiratory tract infections or the common cold in children [28,29,30,31,32]. Among them, however, there are few reports using heat-killed LAB. Some clinical trials using heat-killed LAB were performed in adults [33,34]. Here we conducted a randomized, double-blind, placebo-controlled trial for the prevention of respiratory tract infections in 172 healthy children and clarified that the intake of the heat-killed probiotic strain K15 did not suppress fevers or reduce absences from preschools. However, it is notable that the duration of febrile days was significantly reduced in the group of K15 intake compared to the placebo control group, among children who took less yogurt or fermented foods including LAB, except for test samples. This result indicates that K15 has a beneficial effect just for children who eat less than two fermented food or yogurt items per week. As the intake of other yogurt products or fermented foods was not limited in this trial, such dietary habits have possibly been involved in activating the immune systems in the main, thus masking the apparent effects of K15.

Although there have been many reports on the clinical effects of live probiotic LAB on the duration and symptoms of the common cold in children, the results from these studies differ depending on the study period, as well as the type and dosage of probiotics and the age of subjects [28,29,30,31,32]. Kump et al. [28] reported that in children attending daycare (*n* = 97 in each group), *Lacticaseibacillus rhamnosus* (previously called *Lactobacillus rhamnosus*) GG (LGG) intake for 28 weeks reduced the duration of respiratory symptoms compared to the placebo group but showed no effects on reducing the number of viral findings or the respiratory symptoms. Leyer et al. [29] and Rerksuppaphol et al. [30] performed clinical trials using a combination of *Lactobacillus* and *Bifidobacterium* in children. In both trials, common cold symptoms were significantly suppressed by probiotic intervention. On the other hand, according to the report on a clinical trial in infants, the effect on the suppression of fevers due to common cold symptoms was not clear [31]. In this trial, *Bifidobacterium animalis* subsp. *lactis* and *Lactobacillus rhamnosus* were administered to healthy infants aged 8–14 months in a dose of 10^9^ cfu/day for six months. As the report mentioned above, the results of these clinical trials on the intervention using live probiotics were heterogeneous.

In a meta-analysis of clinical trials using LGG in acute gastroenteritis, it was shown that more than 10^10^ cfu were necessary to improve the duration of diarrhea [35]. Based on these observations, the dosage of LAB might be important to show the potential ability to activate immune functions in clinical trials. However, it is difficult in general to increase the dosage of “live” LAB for infants or children because of the risk of diarrhea and other side effects. Therefore, “heat-killed” LAB are beneficial in terms of the dosage control to exert their probiotic effects.

In our clinical trial, the effects of an oral intake of heat-killed K15 on anti-infection immunity were tested. We focused on the analysis between two subgroups, i.e., with or without the intake habits of fermented foods including other LAB except for in test samples and confirmed that daily supplementation of heat-killed K15 was beneficial to those without the above-mentioned dietary habits. It should be noted that the clinical trials using live probiotics in previous reports were implemented under dietary restrictions, such as other probiotics, microbial medicines or vitamin/mineral supplements [28,29,30].

LAB modulate immune systems through activating innate immune cells. LAB are recognized in the small intestine by DCs and macrophages, which results in activating overall innate immunity including natural killer cells. Moreover, cellular functions of T cells and B cells are subsequently upregulated through a variety of cytokines and co-stimulatory molecules provided by DCs and macrophages; thus, LAB also impact acquired immunity [20,36]. Therefore, at least a part of the health benefit effects of LAB may well be attributed to the activation of the immune system, as it has been described in many clinical trials but required large doses. Instead of taking a large number of probiotic bacteria, heat-killed LAB are safer than live bacteria because of its very low risk of diarrhea resulting from an excess intake [24]. The safety of K15 intake (5 × 10^10^ per day for four months) was confirmed with no differences between the placebo and K15 groups in adverse events.

We previously reported the immunomodulatory effects of heat-killed K15 in human cells regarding its promotion of Th1 cells and IgA production [19,25]. In particular, the level of IgA is an important indicator of immune activation for both innate and acquired immunity. In the present study, the level of the salivary total sIgA concentration in the K15 group was significantly kept higher than that in the placebo group. This result was compatible with our previous in vitro studies, showing that K15 promoted the IgA production from B cells through IL-6 and IL-10 secreted by mDCs [19]. Some reports showed the effects of probiotic bacteria on promoting antibody production, i.e., neutralization antibodies or antigen-specific IgG in human sera [37,38], and these antibodies are important for anti-infection immune responses. Some groups reported the activation of the production of these antibodies by the oral administration of heat-killed LAB in mice [8,39]. Although serum samples were not available in this clinical trial, there is a possibility that K15 intake affected the pathogen-specific IgG or neutralization antibody production in the sera of the intervention group.

In addition to IgA production, type I IFNs are direct mediators of protection against viral infections. We showed that IFN-β production by mDCs occurred in response to heat-killed K15. Moreover, IFN-β promoted IL-12 secretion, resulting in the promotion of Th1 cell differentiation [25]. These sequential responses from innate to acquired immunity work for upregulating anti-infection immune responses [40]. In collaboration with mDCs, pDCs secrete a large amount of type I IFN (IFN-α) and activate innate immune responses [14,15]. We also observed that heat-killed K15 stimulated a robust IFN-α production from human pDCs (unpublished data). These high abilities of K15 to promote type I IFN production from mDCs and pDCs could contribute to anti-infection immune responses, resulting in a reduction of common cold severity or duration.

## 5. Conclusions

Heat-killed probiotic strains of LAB, such as K15, are safe and highly expected to be utilized for the purpose of activating immune systems in infants and children. We concluded that K15 is effective in reducing the risk of viral infections in preschool children, and that it is beneficial to use heat-killed probiotic strains of LAB as food in terms of their safety and efficacy.

## Figures and Tables

**Table 1:** Baseline characteristics.

	K15 (*n* = 87)	Placebo (*n* = 85)	*p* Value
Male/female			0.88
Male, *n* (%)	41 (49.4)	42 (50.6)	
Female, *n* (%)	46 (51.7)	43 (48.3)	
Preschool year			0.92
The 3rd year, *n* (%)	28 (49.1)	29 (50.9)	
The 2nd year, *n* (%)	30 (50.0)	30 (50.0)	
The 1st year, *n* (%)	29 (52.7)	26 (47.3)	
Family history of allergy			
Yes, *n* (%)	63 (49.2)	65 (50.7)	0.48
Consumption of test foods			
Average days (mean ± SD)	105.13 ± 10.08	105.28 ± 10.77	0.50
Intake of foods including lactic acid bacteria			
Average days (mean ± SD)	37.29 ± 32.36	40.92 ± 38.21	0.50
Number of vaccination			0.44
None, *n* (%)	34 (50.5)	33 (49.3)	
1, *n* (%)	11 (64.7)	6 (35.3)	
2, *n* (%)	42 (47.7)	46 (52.3)	
Frequency of common cold symptoms in family			
Average days (mean ± SD)	11.93 ± 19.09	13.01 ± 17.23	0.70

**Table 2:** The number of febrile days during the test period.

	K15 (*n* = 87)	Placebo (*n* = 85)	*p* Value
Average days (mean ± SD)	2.24 ± 2.54	2.67 ± 3.43	0.35

**Table 3:** Secondary outcomes and adverse events.

	K15 (*n* = 87)	Placebo (*n* = 85)	*p* Value
Absence from preschool			
Average days (mean ± SD)	2.14 ± 3.65	2.31 ± 2.96	0.74
Incidence of influenza virus infections			
*n* (%)	14 (16.1)	19 (22.4)	0.34
Febrile days by influenza virus infection			
Average days (mean ± SD)	0.37 ± 0.92	0.52 ± 1.07	0.32
Adverse events, *n* (%)			
Respiratory tract	83 (51.6)	82 (49.4)	0.74
Gastrointestinal tract	46 (28.6)	47 (28.3)	1.00
Others	30 (18.7)	32 (28.3)	0.89

**Table 4:** The number of febrile days in children with a rare intake of other lactic acid bacteria.

	K15 (*n* = 36)	Placebo (*n* = 41)	*p* Value
Average days (mean ± SD)	1.69 ± 2.08	3.17 ± 3.98	0.042

**Table 5:** sIgA concentrations in saliva.

	K15	Placebo	*p* Value
Total sIgA concentrations, mg/dL (mean ± SD)			
Before (*n*)	53.63 ± 42.26 (83)	54.63 ± 50.80 (83)	0.892
After (*n*)	53.31 ± 42.22 (82)	42.82 ± 28.20 (83)	0.063
Change (*n*)	3.20 ± 47.21 (81)	−12.49 ± 51.24 (81)	0.044
